# Nephrotic Syndrome: A Review

**DOI:** 10.7759/cureus.53923

**Published:** 2024-02-09

**Authors:** Priyanshu R Verma, Praful Patil

**Affiliations:** 1 Medicine, Jawaharlal Nehru Medical College, Datta Meghe Institute of Higher Education and Research, Wardha, IND; 2 Microbiology, Jawaharlal Nehru Medical College, Datta Meghe Institute of Higher Education and Research, Wardha, IND

**Keywords:** proteinuria, kidney, deficiency, steroid sensitive, nephrotic syndrome

## Abstract

Nephrotic syndrome (NS) is characterized by hypoalbuminemia, severe proteinuria, and peripheral edema, frequently in conjunction with hyperlipidemia. Individuals usually show symptoms of weariness and swelling, but no signs of serious liver damage or cardiac failure. With characteristic medical symptoms and evidence of hypoalbuminemia and severe proteinuria, NS can be diagnosed.

The majority of NS episodes are classified as unexplained or primary; the most prevalent histopathological subgroups of primary NS in people are focal segmental glomerulosclerosis and membraneous nephropathy. Thrombosis of the veins with high cholesterol levels is a significant NS risk. Acute renal damage and infection are further possible side effects.

The pathobiochemistry of NS involves alterations in genes that affect the selectivity of the kidneys and abnormalities in proteins related to podocytes. Understanding the molecular mechanisms that influence these processes is crucial to developing specific and targeted therapeutic approaches.

The need for invasive renal biopsies throughout the diagnosis process may be lessened by the development of non-invasive nephrotic syndrome biomarkers, such as microRNAs.

Corticosteroids are frequently used as the initial line of defense in NS treatment. However, some individuals need other treatments since a resistant type of NS also exists. The use of calcineurin inhibitors, mycophenolate mofetil, and rituximab is mentioned in the text, along with current research to identify safer and more efficient therapeutic choices.

The complicated kidney condition NS has several underlying causes and symptoms. For the diagnosis of this ailment as well as the creation of focused therapies, an understanding of the pathophysiology and the identification of possible biomarkers are essential.

## Introduction and background

One of the causes of advanced kidney disease is nephrotic syndrome (NS). The primary symptom a physician should suspect in NS is a swollen body as the patient suffers from high blood pressure with hyperlipidemia. Membranous nephropathy, focal segmental glomerulosclerosis, and diabetic nephropathy are frequent causes in adults. Adults often have underlying diseases as the primary cause of many secondary causes. Through serologic testing and renal consulting, the cause of the NS should be identified. For patients whose condition has no recognized origin or classification, a renal biopsy is required. Symptoms, side effects, and the underlying reason are all addressed throughout treatment [1]. A living-donor transfusion might require weeks to months of waiting, instead of years. As increased dialysis duration predicts lower individual and donor mortality following transplantation; less time spent waiting is a significant benefit. Furthermore, as the donor and individual are usually in the same operating room, the organ suffers less ischemia injury. Generally, the kidney condition is better than in healthy donors, causing enhanced performance earlier on and roughly four years of transplant retention. Human cell antigen compatibility is also improved and indicates more accurate outcomes when an alive donor is linked to the individual [2].

According to area and ethnicity, the prevalence of idiopathic NS ranges from 1·15-16·9 per 100,000 children. Although the precise etiology of idiopathic NS is yet unclear, it is thought to be due to immunologic instability, chemicals circulating in our body, or inherited formational anomalies of the podocyte. The prognosis for long-term kidney outcomes is excellent for steroid-responsive illness [3].

Massive proteinuria, hypoalbuminemia, and edema are features of the problematic form of an NS known as congenital nephrotic syndrome (CNS). The major characteristic of CNS is significant plasma protein leakage. Patients may be diagnosed while still in the womb or the first few weeks after birth, usually before the age of three months. Either hereditary or nongenetic etiologies can be linked to CNS etiology. It has been suggested that this condition is caused by pathogenic mutations in NPHS1, NPHS2, LAMB2, WT1, and PLCE1 genes. The clinical course is made more difficult by substantial edema, infections, thrombosis, hypothyroidism, failure to thrive, and other factors. During their first month of life, the mainstays of therapy include gaining vascular access, routine IV albumin infusions, the use of diuretics, infection prevention, and nutritional support. Transplanting a kidney is the most effective treatment for these patients. Clinicians continue to face difficulties with CNS diagnosis and treatment. The pathogenesis, diagnosis, and treatment of CNS patients are now more well-known thanks to a review by AbuMaziad et al. [4].

The kidney illness known as NS, which is common in children, causes protein, fluid, and nutrition loss in the urine, and is characterized by changes in glomerular filtration. Peripheral, gravity-dependent edema affects the majority of patients; nevertheless, severe instances also show anasarca and ascites. Due to the underlying pathology and medications used to treat it, the condition has several long-term consequences, such as hyperlipidemia, metabolic bone disease, and deficits in certain micronutrients. The key to effective management is pharmacological and nutritional therapies. Edema is treated with corticosteroids, albumin, diuretics, sodium, and fluid restriction, and these medications are combined. Patients with recurrent episodes of illness or conditions that are resistant to steroid treatment may benefit from steroid-sparing treatments such as alkylating drugs, calcineurin inhibitors, and dietary changes that exclude dairy and gluten. To provide the best care possible for children with NS, nutrition experts should become familiar with the complexities of managing this condition [5].

## Review

Methodology

We undertook a systematic search through PubMed and PubMed Central in November 2020 using keywords such as "nephrotic syndrome" and "glomerulosclerosis" ((nephrotic syndrome [Title/ Abstract]) OR (NS [Title/ Abstract] OR ("nephrotic syndrome" [MeSH Terms]) AND ("glomerulosclerosis" [Title/ Abstract]) OR (GS [Title/ Abstract]) OR ("glomerulosclerosis" [MeSH Terms])).

**Figure 1:**
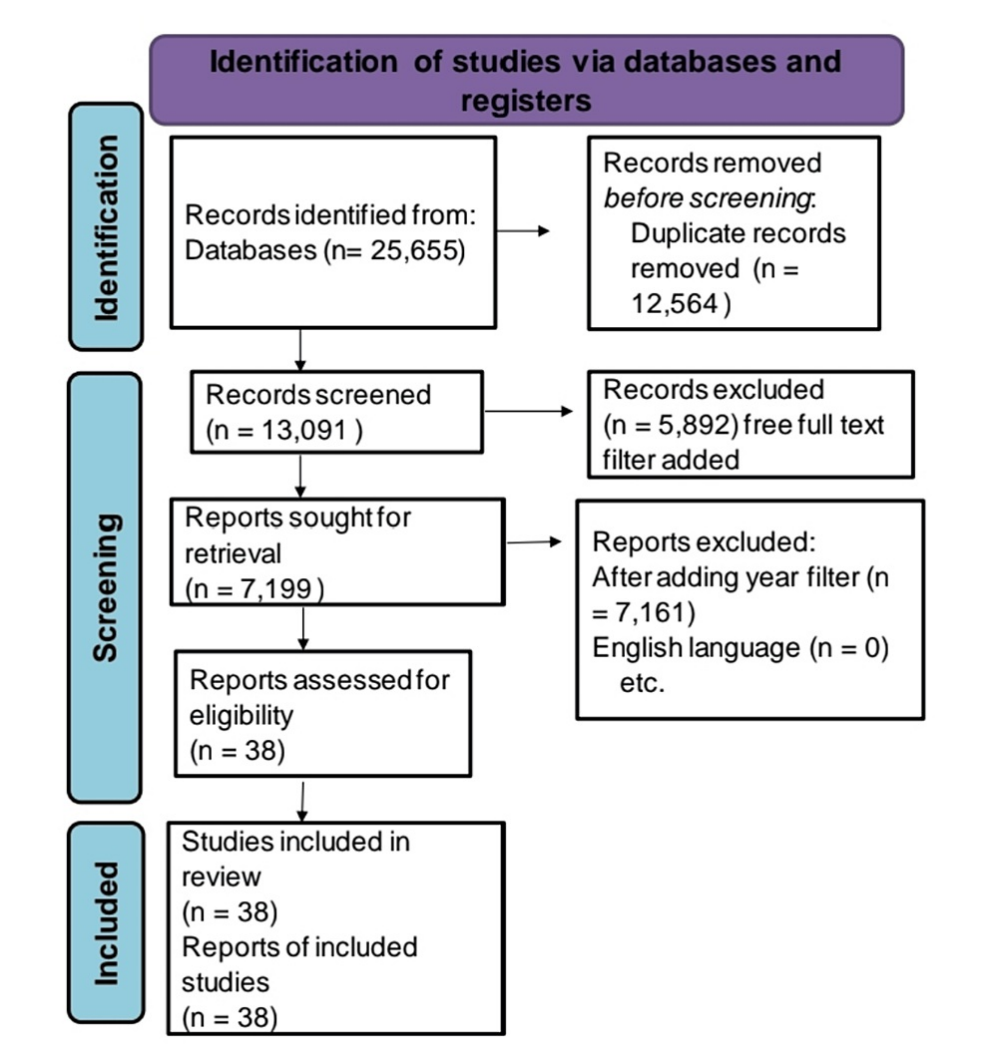
Inclusion and exclusion criteria

We additionally searched for key references from bibliographies of the relevant studies. The search was updated in February 2022. One reviewer independently monitored the retrieved studies against the inclusion criteria, in the beginning, based on the title and abstract and then on full texts. Another reviewer also reviewed approximately 20% of these studies to validate the inclusion of studies (Figure 1).

Pathogenesis and pathophysiology

Urinary protein loss (mostly albumin) is caused by damage to or dysfunction of the glomerulus's structural elements, such as the basement membrane, endothelium surface, or epithelial cells (podocytes). Only some proteins can be excreted in the urine due to restrictions on the size of the pore in the basement membrane and the charges of the barriers involved [1]. Edema, hypoalbuminemia, hyperlipidemia, and protein loss in the urine are all symptoms of NS. The glomerular podocytes are harmed by several disorders, which leads to NS. Together with the endothelial cells of the glomerular capillaries and the basal membrane, these specialized epithelial cells create a filter that traps plasma proteins in the bloodstream. Proteinuria results from a problem with this filter. Primary focal segmental glomerulosclerosis, membranous glomerulonephritis, and minimum change are the three most prevalent primary glomerular disorders. Although the well-known types are uncommon, our knowledge of podocyte function and the etiology of NS have considerably improved as a result of the discovery of important gene abnormalities [6]. There is no clear explanation for how edema develops in NS. Increased glomerular permeability to albumin and other plasma proteins appears to be the main problem. Edema is brought on by raised drainage of liquid in the space between blood vessels via the vascular region mainly related to kidney salt absorption with a lowering of flow due to a decrease of albumin in the bloodstream. Though not fully understood, the biological relation for thrombus formation by normal saline appears to be multifaceted and involves a raised drainage of liquid in the space between blood vessels via the vascular region. A patient's risk of thrombosis is substantially elevated if they also have prothrombotic genetic variants and NS [7].

Pathobiochemistry of NS

The increased permeability of the glomerular capillary wall for macromolecules is the underlying cause of NS. Persistent NS has a poor prognosis, a significant risk of developing end-stage renal failure, and a high risk of cardiovascular problems because of severe hyperlipidemia. It is still unclear what causes increased glomerular permeability in certain glomerular disorders. Recent research has provided fresh insight into the molecular processes of glomerular permselectivity by identifying the mutant genes for a few podocyte proteins in rare familial types of nephrotic disease. As time goes on, it becomes more and more clear that spontaneous mutations of podocyte proteins, such as podocin, may exist in some patients with acquired NS. Podocyte injury that causes either apoptosis or promotion of proliferation and some type of healing, including glomerular sclerosis, may cause the expression of additional podocyte proteins to vary over the course of experimental NS. Clear therapeutic implications could result from a deeper understanding of these systems. Although it is believed that glomerular permeability factors play a role in several non-inflammatory glomerular disorders, their molecular identification is difficult. This is likely due to the nonhomogeneous nature of the underlying diseases. For instance, spontaneous mutations in some podocyte protein genes, increased production of glomerular permeability factor (perhaps by thymus cells), or decreased levels of glomerular permeability factor inhibitors in nephrotic urine may all contribute to the development of localized segmental glomerulosclerosis.

There are distinct differences between the factors that contribute to glomerular sclerosis and those that increase glomerular permeability. Proteinuria does not just appear to result from glomerular injury; it may also damage tubules and start interstitial fibrosis, which would speed up the development of chronic renal failure in proteinuric renal disorders. The latest updates in the knowledge of the mechanisms behind tubular protein reabsorption may provide us with new methods for halting the growth of chronic kidney illness. In patients with severe proteinuria who are resistant to treatment, cubilin inhibitors may be able to reduce tubular and interstitial damage. All patients with prolonged NS should be treated for nephrotic hyperlipidemia, which increases the risk of cardiovascular problems. Prospective controlled trials are still needed to confirm the hypothesized beneficial effect of hypolipidemic medications (namely statins) on cardiovascular risk and chronic renal failure. Recent progress in understanding podocyte biology in uncommon hereditary glomerular disorders has opened up the possibility of understanding the molecular etiology of increased glomerular permeability in the far more commonly acquired forms of NS in the near future [8].

Refractory NS: a rare immune disease

Worldwide, both children and adults are affected by the rare but severe kidney illness known as NS. Its clinical manifestations frequently involve hyperlipidemia and include peripheral edema, severe proteinuria, and hypoalbuminemia. Two to seven per 100,000 children and three per 100,000 adults are the stated annual incidence rates. Even though it only occurs on occasion, it accounts for up to 20% of end-stage renal disease (ESRD) in children and roughly 12% of all ESRD causes. NS can have a variety of etiologies, including secondary illnesses brought on by medications, infections, and neoplasia as well as primary glomerulonephritis. Although there is a lot of evidence that indicates that immune-mediated disease that results in glomerular visceral epithelial cells destructs glomerular filters specifically affected by primary NS, its exact cause is still unknown [9].

Types of primary NS

Primary NS is divided into three main pathophysiological subtypes: focal segmental glomerulosclerosis (FSGS), minimal change disease (MCD), and idiopathic membranous nephropathy (IMN). They all have immunological damage to renal podocytes that are not very inflamed as part of their pathogenic pathways, despite differences in some features [9]. IMN, which is typically categorized as an autoimmune disease, acts as a catalyst in the sub-epithelial layers of the glomerulus and exhibits immune deposition, which causes the destruction of the walls of small blood vessels supplying the functional unit of the kidney [10]. The discovery that anti-podocyte-autoantigen antibodies are present in 5-10% of cases and that podocyte autoantigen detection is in 60-80% of cases was hailed as a revolutionary development in our understanding of the underlying pathomechanism of systemic nephropathy [11,12]. Conventionally, MCD and FSGS were regarded as separate entities. Nevertheless, given that FSGS represents an advanced stage of disease progression than MCD, there is now sufficient data to demonstrate different types of related chronic diseases [13]. It is believed that a general disturbance of T-cell activity is the immunological origin of these problems, bringing on the generation of pro-inflammatory factors or indirectly affecting the glomerulus and its function [14].

Among adults, IMN is the main cause of NS, despite being extremely uncommon in youngsters. Childhood NS is most frequently caused by MCD and FSGS. They account for 10-15% and 40%, respectively, of older instances of NS [9,15].

Biomarkers

Massive proteinuria and low blood protein levels brought on by several disorders, such as minimal change NS (MCNS), FSGS, and membranous glomerulonephropathy, are clinical conditions known as NS. Invasive renal biopsies are generally required to distinguish between diagnoses. In rare instances, it can be challenging to distinguish between MCNS and FSGS even with a biopsy. There is currently no better choice for diagnosis than a kidney biopsy. Non-coding RNA molecules called microRNAs (miRNAs), which manage the removal of specific chromosomes during cell division steps, are found capable of removing the disease-causing, unwanted part from the gene and can be in length up to 20 nucleotides. MiRNAs have been demonstrated to function as non-invasive biological markers of a variety of illnesses, including kidney disorders, in serum and urine. In this paper, we provide a summary of the current understanding of miRNAs as potential NS indicators [16].

Treatment

Corticosteroids are usually prescribed as the first line of treatment for NS. However, 20% of neonates having NS are resistant to steroids, even though the majority of neonates having the condition are steroid-sensitive. In addition, most pediatric children who react to steroids first get worse, and a lot of them do so frequently and require recurrent sessions of steroid therapy, leading to steroid dependence [17]. Therefore, the category of renal disease in sufferers who have become steroid-dependent or resistant with a high rate of recurrence is referred to as refractory NS (RNS) [18]. Children and adolescents with RNS make up approximately 25-40% of instances of NS, while adults with RNS are more likely to develop the condition in up to 70% of cases. For instance, 10-20% of mature MCD cases develop steroid rebellion, and the recurrence rate for MCD is around 50% [9,19,20]. The terms "NS" and "mature kidney disease" are often used interchangeably in journals and medical contexts due to the high occurrence of RNS in adult kidney disease.

Recent years have seen a significant increase in the use of second-line immunosuppressive treatments, such as cytotoxic drugs, calcineurin inhibitors, mycophenolate mofetil, and rituximab, in the treatment of RNS, with encouraging outcomes. However, prolonged utilization of analeptics and some of these immunosuppressants can have substantial negative consequences, including bone loss, lipid disorders, high blood sugar levels, renal toxicity, hypo-immunity, and an increased probability of getting infected. Additionally, these medicines have a poor risk/benefit profile. Severe unremitting edema, significant proteinuria, hypoalbuminemia, and occasionally impaired kidney function are still common in RNS patients. The ineffectiveness of the RNS treatments that are now not used has sparked ongoing research for safer and more efficient alternative therapies. There is a long history of using traditional Chinese medicine (TCM) to treat kidney disease symptoms like proteinuria and edema. TCM has collected a wealth of clinical expertise in treating this condition and has evolved unique views to explain NS through continuous acceptance, different theories, and also experimentation. It is logical that investigations of TCM be undertaken to treat RNS more effectively [9].

General Treatment Measures

Some experts advise that the liquid intake has to be below 1500 ml in 24 hours and dietary salt less than 3 g per day as part of the standard therapy of patients with NS due to the potential pathophysiologic involvement of sodium retention [21].

Treatment of Edema

Even when the glomerular filtration rate is normal, diuretics are ineffective for patients with nephrosis. Because serum proteins are decreased in NS, loop diuretics are less efficient, and patients may need higher dosages than usual. There may be additional diuretic resistance mechanisms. The prolonged duration of action of oral loop diuretics with twice-daily treatment is typically chosen. However, in cases with severe NS and edema, intestinal wall edema may make it difficult for the diuretic to be absorbed through the gastrointestinal tract, necessitating IV diuretics. One to two kilograms of water weight should be achieved in a 24-hour period, with a relatively progressive diuresis controlled by daily weight monitoring. Any acceptable starting dose of furosemide (Lasix) is 40 milligrams taken two times a day or for bumetanide is 1 milligram taken two times a day. If this shows any insufficient progression of dropsy or different signs of pitting swelling, the dose should be roughly doubled every one to three days. The maximum dosage of furosemide is roughly 240 milligrams one time else 600 milligrams once a day. However, this limit is not supported by any convincing evidence or logic. If the patient's clinical reaction is still inadequate, the patient may be treated by switching IV loop diuretics, giving chlorthalidone orally, or starting an IV meal containing 20 percent body protein before starting an IV diuretic meal in the stomach [7].

Dialysis

Hemodialysis (HD) has become more often used in newborns and young children with renal failure in recent years. Even in newborns with CNS nephrectomies, HD is possible. In sufferers having a live kidney transplant, those who reside nearby might receive HD treatment before the kidney transplant (KTx) for a few weeks. Even infants with peritoneal dialysis (PD) therapy issues (infections and technical issues) can benefit from HD [22]. It is the process through which CNS in children may be treated if renal failure develops or if a bilateral nephrectomy is performed. Infants were first given continuous PD in the 1980s [23,24]. Results later got more accurate, and it became clear that infants' non-renal comorbidity is the main risk factor for mortality [25].

Acute kidney injury aggravating the MCD type of NS

Of all cases of NS in children, MCD causes 70% to 90%. Adult individuals with the condition, notably those over 60, also develop NS. A foot-process fusion affects the purification of aqueous and dissolved substances, which causes a moderate alteration in renal function in 20% to 30% of individuals. When proteinuria is remitted, the glomerular filtration rate drops by 20% to 30% and returns to normal. Acute kidney injury has been documented in several publications over the past 50 years, roughly one-fifth to one-third of which occurred in older patients without preexisting or concurrent renal illness. Male preponderance, age > 50, significant protein in the urine, an acute drop of an abnormally small quantity of albumin in the blood, a history of hypertension, injury vessels on renal tissue extraction, and ischemic tubular necrosis are clinical indicators. Acute kidney damage can necessitate dialysis for a few weeks or months until the proteinuria goes away and the oliguria goes away. Renal function may occasionally not improve. A hypothesis has been made to explain tubular cell ischemia necrosis as a result of endothelin-1-induced vasoconstriction occurring at the outset of proteinuria. In individuals with minimally changed illnesses, diuretic-induced hypovolemia and nephrotoxic substances are the main causes of acute kidney injury. Acute renal injury is infrequent in children when there are no concurrent problems. The main risk factors include steroid resistance, nephrotoxic medicine, and infection. Supportive therapy's primary purpose in all patients is to purchase time until glucocorticoids can resolve proteinuria and reverse renal failure [26].

Glomerular filtration barrier

Substantial leaking of albumin present in the blood to urea is a key characteristic of CNS. Most of the time, this occurs due to the changes inside the chromosome, which produce albumin in the glomerular capillary wall and podocytes, which regulate renal cleaning [27,28]. Three layers make up this filter: the glomerular basement membrane (GBM), the fenestrated endothelium, and the epithelial cell layer (podocytes), which has interposed slit diaphragms (SD) and distal foot processes. Water and tiny plasma solutes are typically the only substances that can pass through the barrier because it functions as an efficient size- and charge-selective molecular sieve. Because the GBM and especially SD impede the flow of albumin and other bigger plasma proteins, only a small amount of protein is present in the ultrafiltrate that reaches the Bowman space. However, it is now understood that proteinuria can result from a fundamental abnormality in either the SD or GBM. The GBM's function in the renal ability of a membrane to discriminate between anions and cations has been challenged in recent times [29].

The GBM is a well-known protein network made up of negatively charged proteoglycans, type IV collagen, laminin, and nidogen. Elsewhere, SD's exact shape of the molecule could be a matter of debate. The foundation of SD is likely made up of recently discovered foot-like cells present in the kidney having proteins like FAT2, dendrin, FAT1, Neph2, nephrin, and Neph1 [29,30]. The adapter proteins podocin, cortactin-CD2-associated protein (CD2AP), zonula occludens-1 (ZO-1), calcium/calmodulin-dependent serine protein kinase (CASK), and membrane-associated guanylate kinase (MAGI-1), which are present inside foot-like cells present in the kidney, interact with these proteins and form extracellular associations with one another. These participate in cell signaling through the slits in the membrane region to foot processes by connecting to the actin cytoskeleton of the podocyte cells. The actin network and its connecting proteins, like actinin-4, are essential for maintaining the podocyte's intricate structure. It's interesting to note that effacement of the podocyte foot processes results from a disruption of that molecule group in the central nervous system with some illnesses having protein present in urine [29].

CNS

Infantile NS (INS), which develops during the early one to two years as typically has been earlier found, is distinguished from CNS, which starts showing symptoms within the initial three months [22]. CNS of the Finnish type (CNF) can be characterized by high levels of proteinuria, severe hypoproteinemia, edema, and subsequent symptoms brought on by high levels of proteinuria found soon after birth. There have been reports of familial and occasional CNF cases in newborns in Finland without Finnish heritage during the 1900s, which suggests that CNF had not been considered during those days [31]. The care of CNF is difficult even now even after the availability of advanced medical equipment. Since there is varying seriousness for the illness, patients having central nervous system complications have received treatment over the last two to three decades using a variety of protocols [22,32]. Due to the rarity of the central nervous system and its genetic makeup, there is insufficient evidence to establish a single therapy strategy for all CNS patients. The purpose of this study is to justify the timing and rationale of early aggressive treatment, which consists of improved nutrition, albumin inoculation, rapid bilateral nephrectomy, dialysis, and KTx at one to two years of age [22].

Steroid-resistant NS (SRNS)

After receiving daily corticosteroid therapy, more than 85% of children and adolescents with idiopathic NS (with the majority between one and 12 years old) exhibit complete remission of proteinuria. SRNS is a condition in which patients do not demonstrate remission following a four-week course of daily prednisolone therapy. The majority of patients' renal histology reveals the presence of FSGS, MCD, and (occasionally) mesangial proliferative glomerulonephritis. One of the essential podocyte genes has mutations in one-third of SRNS patients. The unknown moving component could generally be responsible for the rest of the cases of SRNS. It is advised that patients with hypertension receive further treatment with medications that block the renin-angiotensin axis to lower any lingering proteinuria. The last stage of kidney failure is a possibility for SRNS individuals who do not improve after being treated with immunosuppressive medications. These patients might also have deteriorating renal function. A third of the individuals who obtain a renal transplant have recurrent FSGS in the allograft, and rituximab, blood exchange, and increased immunosuppressive agents have been utilized in conjunction to treat this illness [33].

NS in pregnancy

It is quite uncommon for NS to manifest during pregnancy. According to reports, 0.028% of pregnancies result in primary renal disease-related nephrosis [34]. Because it can be challenging to distinguish from preeclampsia and because the two conditions may coexist, the incidence of NS secondary to primary glomerular disease is unknown without a histologic diagnosis. Elevated creatinine is a known risk factor for unfavorable pregnancy outcomes [35,36]. Additionally, the course of the mother's kidney illness may be sped up during pregnancy. The impact of NS on pregnancy outcomes in the absence of substantial renal impairment is less apparent. Women with NS alone who don't have substantial hypertension or renal insufficiency tend to do well. The literature contains scant information on this particular subset of patients. Knowing how these patients' clinical outcomes developed will help with NS counseling and direct the care of these high-risk pregnancies [34,37,38].

Table 1 provides a summary of the articles included.

**Table 1:** Summary of the articles included in the review CNS: congenital nephrotic syndrome; NS: nephrotic syndrome; IMN: idiopathic membranous nephropathy; FSGS: focal segmental glomerulosclerosis; MCD: minimal change disease; RNS: refractory nephrotic syndrome; PD: peritoneal dialysis; GBM: glomerular basement membrane; SD: slit diaphragms; CNF: congenital nephrotic syndrome of the Finnish type

Author	Year	Findings
Politano et al., [1]	2020	Edema and high blood pressure in older adults; various causes require testing, nephrologist consultation, and possible kidney biopsy for diagnosis and treatment.
Warady et al., [2]	1997	NS, characterized by excessive proteinuria, edema, hypoalbuminemia, and hyperlipidemia, is linked to various illnesses, necessitating prompt diagnosis and treatment to address the underlying systemic disorder.
Noone et al., [3]	2018	Idiopathic NS is prevalent in children, affecting a large number of them, caused by immunological dysregulation, systemic substances, or podocyte defects, with excellent long-term kidney outcomes.
AbuMaziad et al., [4]	2021	The pathogenesis, diagnosis, and treatment of CNS patients are now more well-known thanks to this review
Hampson et al., [5]	2021	To provide the best care possible for children with NS, nutrition experts should become familiar with the complexities of managing this condition
Walz et al., [6]	2003	NS, characterized by edema, hypoalbuminemia, hyperlipidemia, and protein loss in urine, is caused by disorders affecting glomerular podocytes, resulting in proteinuria.
Bridwell et al., [7]	2021	Edema in NS is caused by increased glomerular permeability to albumin and plasma proteins, raised liquid drainage, and increased risk of thrombosis in patients with prothrombotic genetic variants.
Tesar et al., [8]	2003	Recent advances in podocyte biology offer hope for understanding and treating NS, a condition characterized by increased glomerular capillary permeability, potentially causing end-stage renal failure and cardiovascular issues.
Wang et al. [9]	2018	Nephrotic syndrome is a severe kidney condition affecting both children and adults, characterized by hyperlipidemia and peripheral edema, potentially due to immune-mediated factors affecting the glomerular filter.
Ronco et al. [10]	2012	IMN, an autoimmune disease, causes immune deposition and activation in the glomerular space, disrupting its capillary wall.
Beck et al. [11]	2009	The identification of podocyte autoantigens and their associated autoantibodies has significantly enhanced our comprehension of systemic nephropathy.
Meyer-Schwesinger et al. [12]	2015	The comprehension of podocyte autoantigens and the corresponding autoantibodies has significantly enhanced our knowledge of systemic nephropathy.
Maas et al. [13]	2016	FSGS and MCD are two related, not dissimilar, chronic kidney diseases.
Fogo et al. [14]	2015	T-cell disruption is associated with glomerular function and immunological problems.
Eddy et al. [15]	2003	Adults have IMN primarily; children with nephrotic syndrome often have MCD and FSGS.
Tsuji et al. [16]	2020	Membranous glomerulonephropathy, FSGS, and MCD are all parts of the nephrotic syndrome. Possible diagnostic indicators for miRNAs were considered.
Zhao et al. [17]	2015	Nephrotic syndrome is treated with corticosteroids, but 20% of children are steroid-resistant, and 80-90% relapse, leading to recurrent therapy and steroid dependence.
Saito et al. [18]	2004	Therefore, the category of renal disease sufferers who have become steroid-dependent or resistant with a high rate of recurrence is referred to as RNS.
Gibson et al. [19]	2016	NS affects 25-40% of children and adolescents, with adults more likely to develop the condition, with recurrence rates around 50% for mature MCD cases.
Vivarelli et al. [20]	2017	About 25–40% of children and adolescents have the illness known as NS, which is more common in adults. Recurrence rates for mature MCD cases are approximately 50%.
Huli et al. [21]	2008	The NS treatment should be limited to less than 1500 ml of fluid and less than 3 g of salt.
Hölttä et al. [22]	2020	Hemodialysis is becoming increasingly prevalent in young children, even newborns, who are suffering from renal failure.
Oreopoulos et al. [23]	1979	The process that might require the treatment of CNS in children if renal failure develops or if a bilateral nephrectomy is performed. Infants were first given continuous PD in the 1980s.
Hölttä et al. [24]	1997	Hemodialysis is recommended for children with CNS failure or nephrectomy who require treatment of the kidneys.
Ledermann et al. [25]	2000	Results later got more accurate, and it became clear that infants' non-renal comorbidity is the main risk factor for mortality.
Meyrier et al. [26]	2018	MCD, common in children, can lead to acute kidney injury, more so in older adults. Prompt treatment is vital.
Jalanko et al. [27]	2003	Mutations in genes governing glomerular capillary proteins cause albumin leakage in CNS.
Jalanko et al. [28]	2009	Kidney filtration consists of GBM, endothelium, and podocytes with slit diaphragms.
Habib et al. [29]	1993	GBM and SD have vital proteins; their disruption causes proteinuria.
Pätäri-Sampo et al. [30]	2006	GBM is composed of proteoglycans, type IV collagen, laminin, and nidogen. SD consists of various proteins, including nephrin.
Mahan et al. [31]	1984	CNF presents with high proteinuria, hypoproteinemia, and edema in newborns.
Kovacevic et al. [32]	2003	Treatment of CNF is challenging due to varying severity; multiple protocols used over decades.
Tullus et al. [33]	2018	NS in children: steroids work, genetic mutations in steroid-resistant cases. Calcineurin inhibitors for non-genetic cases; recurrent post-transplant treated with plasma exchange and immunosuppression.
Studd et al. [34]	1969	NS is rare during pregnancy, with 0.028% of pregnancies resulting in primary renal disease-related nephrosis, according to reports.
Hayslett et al. [35]	1994	The incidence of NS secondary to primary glomerular disease is unknown without a histologic diagnosis, but elevated creatinine is a known risk factor for unfavorable pregnancy outcomes.
Nevis et al. [36]	2011	Without a histologic diagnosis, the frequency of NS related to primary glomerular disease is unclear, but high creatinine is an established risk factor for poor pregnancy outcomes.
Lamkee et al. [37]	1961	The study reveals that kidney disease (NS) can accelerate pregnancy outcomes, but its impact on women without significant renal impairment is less clear, requiring further research.
De Castro et al. [38]	2017	The findings of the study indicate that kidney disease (NS) can hasten the course of pregnancy; however, the effects on women who do not have substantial renal impairment are less evident and call for more investigation.

## Conclusions

Proteinuria, or an excessive loss of protein in the urine, is a symptom of NS, which also involves various secondary alterations in the body's fluid, lipid, and coagulation balance. NS has a broad span of etiologies that can affect people of various ages, from congenital origins in childhood to acquired reasons in later adulthood. Relapses and/or steroid dependence, two challenging symptoms to treat, are more common in steroid-sensitive NS (SSNS) patients. Mycophenolic acid and calcineurin inhibitors (CNIs) have been found effective in lowering SSNS relapses. Rituximab is also necessary, although numerous questions regarding the first dosage, course repetitions, and long-term negative effects are still unresolved. SRNS may lead to chronic renal dysfunction, particularly if the condition is resistant to treatment.
